# Editorial: Childhood Diabetes in Low- and Middle-Income Countries

**DOI:** 10.3389/fendo.2021.830700

**Published:** 2022-01-19

**Authors:** Chunxiu Gong

**Affiliations:** Department of Endocrinology, Genetics and Metabolism, Beijing Children′s Hospital, Capital Medical University, National Center for Children′s Health, Beijing, China

**Keywords:** children, diabetes, low- and middle-income countries, status, pathogenesis

A century ago, type 1 diabetes mellitus (T1DM) was almost fatal. Since the discovery of insulin by Banting and Best in 1921, T1D became a chronic condition, but still requiring lifelong treatment. A century later, we have gained a deeper understanding of the disease and increased accessibility to anti-diabetic advancements such as insulin analogs, continuous subcutaneous insulin infusion, and continuous glucose monitoring devices.The quality of life of patients has been greatly improved, but many challenges remain (1). Whether or how these advanced technologies help improve glycemic control needs to be ensured, especially in low- and middle-income Countries. The incidence of T1D in children increased significantly in developing countries (2–5), so did the incidence rate of type 2 diabetes with the growing prevalence of obesity (6, 7). Low- and middle-income countries are facing bigger challenges in disease prevention and control (8). Nowadays, many studies aim to explore the clinical status of diabetes care and the related etiology.

The objective of the Research Topic “*Childhood Diabetes in Low- and Middle-Income Countries: Progress, Challenges and Actions Needed*” was to gather original research articles illustrating the recent advances concerning the diabetes in Low- and Middle-Income Countries. This Research Topic consists of seven original articles.

Two original articles investigated the features, treatment status and glycemic control of children with T1DM in China. Hou L. et al. investigated the features and treatment status of children with T1DM from 33 medical centers in China, and found that the patients were still predominantly receiving multiple daily subcutaneous injections of insulin. The proportion of patients using insulin pumps was only 15.21%, much lower than that reported for developed countries. The blood glucose monitoring was also insufficient. It has been reported continuous subcutaneous insulin infusion (CSII) was not superior to MDI on the glycemic control in long-term follow up.In low- and middle-income countries like China, medical insurance companies don’t pay the costs of the insulin pump, so insulin injection is a cost-effective therapy for Chinese children. Chen X L et al. reviewed publications on HbA1c concentrations in patients aged less than18 years which showed that the glycemic control of children with DM improved during the last decade in east China, but even in high-income countries, there were still 67.6% children with DM whose HbA1c levels are suboptimal. Although accessibility to anti-diabetic advancements such as insulin analogs, CSII, and continuous glucose monitoring devices, have increased, huge efforts are needed to optimize the glycemic control in childhood diabetes all over the world. It also pushes us to develop comprehensive methods in addition to the advanced glycemic control instrument in use.

Qingdao is one of the several cities in China to bring the insulin pumps into reimbursement policy for T1DM in adolescents and children. Hu et al. conducted a retrospective study to evaluate the clinical and economic consequences of continuous subcutaneous insulin infusion (CSII) vs. multiple daily injections (MDI) in children and adolescents with T1DM in Qingdao, they found CSII was more cost-effective than MDI based on the health economics model and CORE mode. However, these results were based on the simulation model with no real ending to support it, and therefore should be interpreted prudently. Additionally, the health economic analysis of T1DM in this study was mainly referred to the published direct medical cost report of T2DM related complications, due to the lack of research data on the treatment cost of T1DM related complications in China. Thus, further studies were needed in the future.

One article studied the misdiagnosis rate of T1DM in Asian cohorts. The prevalence of misdiagnosis has been reported in North-American and European children (9, 10), not in Asian children. However, the high frequency of DKA has been observed in some low- and middle-income countries (11), for which misdiagnosis maybe the reason. Mavinkurve M. et al. conducted a retrospective study of children with T1DM below 18 years of age between January 1st 2010 and December 31st 2019 in Malaysian. Total 119 children (53.8% female) were recruited, 38.7% of cases were misdiagnosed, of which respiratory illnesses were the most common (37.0%) misdiagnosis. The rate of misdiagnosis remained the same over the 10 year period. It occurred more frequently in children <5 years of age. Misdiagnosed cases are at a higher risk of presenting in DKA with increased risk of ICU admission and more likely to have had prior HCP (healthcare professionals) contact. Awareness of T1DM amongst healthcare professionals is crucial for early identification and prevention of DKA, and reducing rates of misdiagnosis.

China has the largest number (116.4 million) of adults with diabetes in the world, however, little is known about the quality of life of patients related to the disease. Long E W et al. described and compared the health-related quality of life (HRQoL) among 403 respondents with diabetes, 404 with prediabetes, and 398 with normal blood glucose. HRQoL declined gradually from the prediabetic population, population with normal glycemic levels to diabetic population. Pain/discomfort and anxiety/depression was more common in normal glycemic population than prediabetic population, so the tool used in the research might not be specific for the population with or without diabetes. The more sensitive and accurate tool is needed to assess the quality of life in low- and middle-income countries.

Usually, demographic information, including age, perinatal risk factors, family history, ethnicity, and clinical manifestation, obesity, metabolic syndrome components, are risk factors for type 2 diabetes mellitus (T2DM), which are also helpful in diagnosing T2DM. Recently, genome-wide association studies have identified hundreds of single nucleotide polymorphism (SNPs) associated with T2DM, and it also has been reported a high genetic risk score (GRS) was associated with a younger age at the time of T2D diagnosis (12, 13). One article by Miranda-Lora et al. investigated whether GRS that combines 10 SNPs could improve prediction models for pediatric onset T2DM. The results showed GRS had a significant association with pediatric-onset T2D (OR = 1.3 per risk allele; p = 0.006). The GRS, clinical risk factors, and GRS plus clinical risk factors had an AUC of 0.66 (95% CI 0.56–0.75), 0.72 (95% CI 0.62–0.81), and 0.78 (95% CI 0.70–0.87), respectively (p < 0.01). GRS improved the predictive properties modestly in pediatric-onset T2D in Mexicans. However, clinical factors, have the highest predictive utility in this population. The control group in this study included normal children. The differential diagnosis between T1DM and T2DM is a big challenge for doctors, so further studies with T1DM patients as the control group are needed.

Obesity has become a serious public health problem worldwide. Gut microbiota dysbiosis has been proposed as an etiologic factor underlying metabolic disease associated with insulin resistance (IR) all over the world including low- and middle-income countries. Little is known about the characteristics of the gut microbiota in obese children. Yuan X et al revealed the different intestinal flora of gut microbiota in obese Chinese children with or without IR. In the IR subjects, they observed a reduction of Firmicutes and an increase of Bacteroidetes. In the insulin sensitive (IS) group, they found Coriobacteriales, Turicibacterales, Pasteurellales and family Turicibacteraceae were more abundant. The correlation between gut microbiota and serum ANGPTL4, adropin indicated gut microbiota may be involved in the regulation of glucose metabolism in obesity.

Although great improvements have been acquired on the management and research about the diabetes in low- and middle-income countries, patients have limited abilities to access the advanced technology. Backward technology does not equal poor glycemic control. We should adjust measures to local conditions, make use of available technologies, and strengthen patient education, so as to improve glycemic control and prognosis. More efforts are needed to care for the diabetic children.

## Author Contributions

The author confirms being the sole contributor of this work and has approved it for publication.

## Conflict of Interest

The author declares that the research was conducted in the absence of any commercial or financial relationships that could be construed as a potential conflict of interest.

## Publisher’s Note

All claims expressed in this article are solely those of the authors and do not necessarily represent those of their affiliated organizations, or those of the publisher, the editors and the reviewers. Any product that may be evaluated in this article, or claim that may be made by its manufacturer, is not guaranteed or endorsed by the publisher.
